# Progress in HAXPES performance combining full-field *k*-imaging with time-of-flight recording

**DOI:** 10.1107/S1600577519012773

**Published:** 2019-11-01

**Authors:** K. Medjanik, S. V. Babenkov, S. Chernov, D. Vasilyev, B. Schönhense, C. Schlueter, A. Gloskovskii, Yu. Matveyev, W. Drube, H. J. Elmers, G. Schönhense

**Affiliations:** a Institut für Physik, Johannes Gutenberg Universität Mainz, D-55099 Mainz, Germany; b Department of Bioengineering, Imperial College London, UK; c DESY Photon Science, Notkestrasse 85, 22607 Hamburg, Germany

**Keywords:** HAXPES, time-of-flight microscope, X-ray photoelectron diffraction, Brillouin zone, *k*-space

## Abstract

An alternative approach to hard-X-ray photoelectron spectroscopy (HAXPES) has been established. The instrumental key feature is an increase of the *dimensionality* of the recording scheme from 2D to 3D. A high-energy momentum microscope detects electrons with initial kinetic energies up to 8 keV with a *k*-resolution of 0.025 Å^−1^, equivalent to an angular resolution of 0.034°. A special objective lens with *k*-space acceptance up to 25 Å^−1^ allows for simultaneous full-field imaging of many Brillouin zones. Combined with time-of-flight (ToF) parallel energy recording this yields maximum parallelization. Thanks to the high brilliance (10^13^ *h*ν s^−1^ in a spot of <20 µm diameter) of beamline P22 at PETRA III (Hamburg, Germany), the microscope set a benchmark in HAXPES recording speed, *i.e.* several million counts per second for core-level signals and one million for *d*-bands of transition metals. The concept of tomographic *k*-space mapping established using soft X-rays works equally well in the hard X-ray range. Sharp valence band *k*-patterns of Re, collected at an excitation energy of 6 keV, correspond to direct transitions to the 28th repeated Brillouin zone. Measured total energy resolutions (photon bandwidth plus ToF-resolution) are 62 meV and 180 meV FWHM at 5.977 keV for monochromator crystals Si(333) and Si(311) and 450 meV at 4.0 keV for Si(111). Hard X-ray photoelectron diffraction (hXPD) patterns with rich fine structure are recorded within minutes. The short photoelectron wavelength (10% of the interatomic distance) ‘amplifies’ phase differences, making full-field hXPD a sensitive structural tool.

## Abstract 2

A highly effective way to cope with the weak signals in hard X-ray angular-resolved photoelectron spectroscopy is introduced. Full-field momentum imaging combined with time-of-flight parallel energy recording constitute a 3D recording scheme, gaining two orders of magnitude in detection efficiency.

## 1. Introduction   

Owing to the increased inelastic mean-free path, angle-resolved photoelectron spectroscopy (ARPES) in the X-ray range is rapidly gaining importance for electronic structure analysis of solids. Pioneering work (Claesson *et al.*, 2004 ▸; Kamakura *et al.*, 2004 ▸; Sekiyama *et al.*, 2004 ▸; Plucinski *et al.*, 2008 ▸; Papp *et al.*, 2011 ▸) revealed the potential of the method and elucidated the role of direct transitions and phonon scattering. Today, instruments for soft X-ray ARPES exist at several synchrotron radiation sources and have reached very high performance. Excellent results are being published with an increasing pace (*e.g.* Xu *et al.*, 2015 ▸; Lv *et al.*, 2015 ▸, 2017 ▸; Lev *et al.*, 2018 ▸; Cancellieri *et al.*, 2016 ▸). In the hard X-ray range the situation is different because rapidly dropping subshell cross sections and increasing background due to phonon scattering make hard X-ray ARPES (HARPES) a difficult task. The pioneering experiment was performed in 2011 by Gray *et al.* (Gray *et al.*, 2011 ▸) and the method was successfully applied to dilute magnetic semiconductors (Gray *et al.*, 2012 ▸; Keqi *et al.*, 2018 ▸; Nemšák *et al.*, 2018 ▸; Sato *et al.*, 2018 ▸; Kobayashi *et al.*, 2014 ▸).

Hard X-ray photoemission is characterized by an information depth of >10 nm which has been exploited intensively in angular-integrating work (see recent overviews by Fadley & Nemšák 2014 ▸; Woicik, 2016 ▸). With increasing probing depth, the relative contribution of the surface is decreasing. HARPES can access genuine bulk bands, in particular for samples with reactive or unprepared surfaces. Hard X-ray beamlines with excellent resolution in the 50 meV range pave the way towards band mapping of capped surfaces, buried layers or interfaces in thin-film devices as well as *in situ* and *in operando* devices. Such exciting prospects break old paradigms of photoemission and raise fascinating future perspectives.

However, HARPES faces serious basic and technical obstacles. *Photoemission cross sections* drop rapidly with excitation energy above photoionization thresholds (Trzhaskovskaya & Yarzhemsky, 2018 ▸), which is relevant not only for valence-band studies but also for core-level spectroscopy. Alongside this drop of signal intensity, the contribution of *electron–phonon scattering* increases strongly and causes a quasi-elastic background that eventually dominates direct interband transitions. The phonon-scattering contribution is reduced at low sample temperatures. Quantitatively, the Debye–Waller factor and the atomic mass of the material specify the fraction of non-scattered electrons and therefore constitute criteria for the feasibility of HARPES for different elements (Papp *et al.*, 2011 ▸). At multi-keV energies, electron wavelengths are much smaller than interatomic distances, wherefore *X-ray photoelectron diffraction* (XPD) plays a significant role. On the one hand, XPD can yield important structural information, as discussed in comprehensive work (for reviews, see, for example, Fadley, 2010 ▸; Woodruff, 2010 ▸; Osterwalder, 2011 ▸; Matsui *et al.*, 2014 ▸). On the other hand, valence-band photoelectron diffraction hampers quantitative band mapping because strong diffraction signatures can modulate the band-structure patterns (Schönhense *et al.*, 2018*a* ▸). With increasing photon energy, the *transfer of photon momentum to the photoelectron* becomes significant. In the hard X-ray range, the resulting shift of the *k*-patterns exceeds the size of a typical Brillouin zone (BZ), an important fact for the interpretation of HARPES data.

The low photoemission cross sections call for optimized electron analyzers. There is a fundamental difference between low- and high-energy ARPES. In the VUV spectral range the performance of an ARPES setup is limited by the maximum count rate of the electron detector. High parallelization on the detector side has led to impressive recording speeds (Babenkov *et al.*, 2015 ▸). At the same time, optimized beamline–instrument combinations offer excellent energy and angular resolution (Hoesch *et al.*, 2017 ▸). However, in the hard X-ray range, the detector is no longer the bottleneck in valence band and shallow core-level photoemission, but the *phase-space acceptance of the analyzer* becomes the crucial criterion. Wide-angle entrance lens optics in front of hemispherical analyzers have reached large angular acceptance of up to ±30° in the tender X-ray range (Keqi *et al.*, 2018 ▸; Nemšák *et al.*, 2018 ▸), recorded along one line in *k*-space. A new type of entrance optics can convert the angular pattern into a *k*-pattern (of variable magnification) at the analyser entrance, thus giving access to very large angular acceptance (Tesch *et al.*, 2018 ▸).

In this paper, we introduce an alternative way to significantly enhance the photoemission signal strength in the hard X-ray range. Instead of an angular-resolving detection scheme, we employ full-field imaging of the photoelectron momentum distribution in combination with time-of-flight parallel energy recording. First experiments have been performed at the new beamline P22 (Schlueter *et al.*, 2019 ▸) of PETRA III (DESY, Hamburg) yielding high photon fluxes in a spot of ∼15 µm × 15 µm. Bandwidths of 630 meV using a Si(111) monochromator crystal (*E*/Δ*E* ≃ 8 × 10^3^, flux 1.1 × 10^13^ *h*ν s^−1^), 140 meV at *h*ν = 5 keV using a Si(311) crystal (*E*/Δ*E* ≃ 3.6 × 10^4^, flux 2 × 10^12^ *h*ν s^−1^) and 47 meV at *h*ν = 5.977 keV using a Si(333) crystal (*E*/Δ*E* ≃ 1.7 × 10^5^, flux 5 × 10^11^ *h*ν s^−1^) provide excellent conditions for testing the new method.

The instrumental key feature is an increase of the *dimensionality* of the recording scheme from 2D to 3D. Common HARPES hemispherical analyzers simultaneously record a certain energy band (typically 10% of the pass energy) and the angular distribution along one line in the emission pattern [*I*(*E*_kin_, θ) recording scheme]. The present technique is based on full-field imaging of the 2D (*k*_*x*_, *k*_*y*_) momentum distribution, as described by Tusche *et al.* (2015 ▸). Instead of energy-filtering using a dispersive analyser, we implement time-of-flight (ToF) parallel detection of many energies [*I*(*E*_kin_, *k*_*x*_, *k*_*y*_) recording scheme]. A low-energy version of this method recently proved high performance in the soft X-ray range (Medjanik *et al.*, 2017 ▸). The new high-energy optics records initial kinetic energies up to 8 keV. The field-of-view in *k*-space was increased by almost one order of magnitude in comparison with the previous low-energy instruments (Tusche *et al.*, 2015 ▸; Medjanik *et al.*, 2017 ▸) by a modified lens design optimized for low aberrations at high initial kinetic energies.

## 2. *k*-imaging ToF HAXPES   

### 2.1. ToF versus dispersive energy recording   

**Figure 1:**
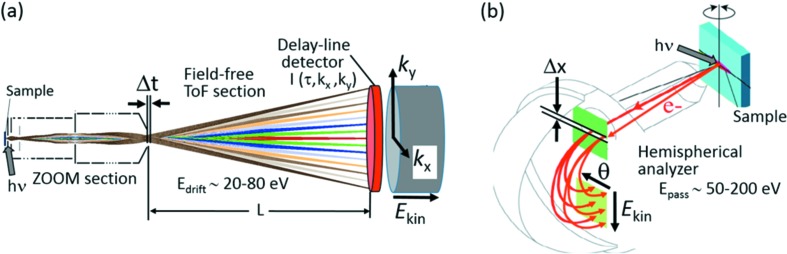
Recording schemes of a HAXPES experiment using a *k*-imaging time-of-flight spectrometer (*a*) in comparison with a dispersive hemispherical spectrometer (*b*). In (*a*), 3D data arrays *I*(*E*_kin_,*k*_*x*_,*k*_*y*_) are recorded using a delay-line detector. There is no beam-confining slit (except the field aperture selecting the region of interest in real space). In (*b*), 2D arrays *I*(*E*_kin_,θ) are recorded and the second angular coordinate is varied either by sample rotation or by deflectors in the lens optics. The desired energy resolution demands a sufficiently ‘thin’ isochrone surface with small width Δ*t* at the entrance of the ToF section (*a*) and a sufficiently narrow entrance slit with width Δ*x* at the entrance of the hemisphere (*b*).

Besides the ‘intrinsic’ obstacles (low cross section, strong phonon scattering, diffraction), the strong retardation of the fast photoelectrons to the pass energy of the analyser constitutes a technical problem which limits performance. This can most readily be seen from Liouville’s theorem, describing the conservation of the phase-space volume in particle optics. The three relevant phase-space ‘coordinates’ are electron velocity (proportional to 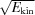), lateral size of the beam (quantified by the magnification *M*) and angular spread α measured with respect to the optical axis (quantified by sinα),
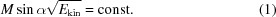
The energy resolution of a dispersive analyser is determined by the size of the entrance slit and the pass energy, *cf*. Fig. 1(*b*) ▸. Assuming a radius of the central beam of 200 mm, an energy resolution of 125 meV (typical for HARPES experiments) requires combinations of pass energy and slit width in the range 30 eV/2 mm to 200 eV/0.25 mm. As a typical example, we consider a deceleration from 6.5 keV to 100 eV, the square root of the ratio yielding a factor of 8. According to equation (1), the energy reduction must be paid for by an increase of the beam size by a factor of 8. In other words, the accepted area on the sample surface is an order of magnitude smaller than the width of the entrance slit.

**Figure 2:**
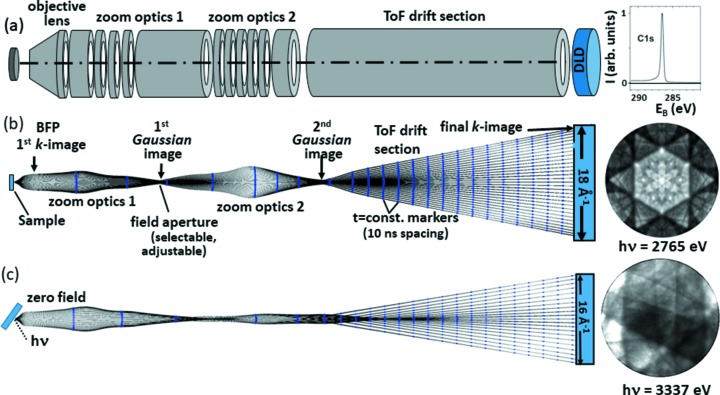
Schematic view (*a*) and simulated electron trajectories (*b*,*c*) for the high-energy time-of-flight momentum microscope. Simulations for an initial kinetic energy of *E*_kin_ = 4 keV with an extractor field of 1 kV mm^−1^ (*b*) and *E*_kin_ = 8 keV at extractor field zero (*c*). The drift energy is 60 eV. Despite zero extractor field in (*c*) the *k*-image diameters are quite similar, 18 Å^−1^ (*b*) and 16 Å^−1^ (*c*). Three lens groups focus and decelerate the beam; an adjustable field aperture in a real image plane can serve for selection of the region of interest. Despite the large *k*-fields the isochrones surfaces are almost planar; blue dots are time markers (10 ns spacing). The right-hand side shows measured results for a graphite single crystal: a typical ToF spectrum of the carbon 1*s* core-level signal (*a*) and two Kikuchi-type photoelectron diffraction patterns taken near normal emission (*b*, detail of a larger pattern) and 30° off-normal (*c*).

Imaging ToF spectrometers (demanding pulsed excitation) do not require slits but instead require an isochrone surface with narrow width Δ*t* at their entrance, see Fig. 1(*a*) ▸. Energy resolution d*E* and time-of-flight τ are determined by the drift energy *E*_d_ in the ToF section (of length *L*) and the time resolution dτ of the detector (Oelsner *et al.*, 2001 ▸; Schönhense *et al.*, 2001 ▸),
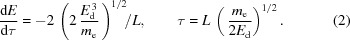
Besides *L*, these equations do not contain any geometry factors (*m*_e_ is the free-electron mass). For the instrument used in this work (*L* = 900 mm, detector diameter = 80 mm, dτ < 200 ps), nominal resolutions between 180 meV and 23 meV are reached at drift energies between 80 and 20 eV. The best energy resolution reached so far was 17 meV (using the identical detector in a similar setup). There are no restrictions in beam size or angular spread, as long as the isochrone surfaces in the ToF section are sufficiently planar (*cf*. Fig. 2 ▸ for simulations of the correct lens geometry). In practice, a constant image-field curvature due to the planar detector and an additional intensity-dependent curvature due to the space-charge effect can be corrected as described by Schönhense *et al.* (2018*b* ▸). The phase-space confinement induced by the entrance slit of a hemispherical analyzer is thus replaced by the demand for sufficiently precise timing conditions of the source (for PETRA III pulse widths of ∼50 ps r.m.s.) and detector [delay-line detector (DLD) resolution ≃ 150 ps (Oelsner *et al.*, 2001 ▸)].

At this point we conclude that, for pulsed excitation sources, Liouville’s theorem is in favor of imaging ToF instruments, owing to their linear columns lacking any slits. The principal advantage of ToF over dispersive energy recording is most apparent when aiming at very large fields-of-view at high energies [leading to huge phase-space volumes, equation (1)]. The same argument holds when aiming at high energy resolution in the few 10 meV range, demanding a very small entrance slit in a hemispherical analyzer. Bandwidths of <20 meV have been reached in several soft X-ray beamlines (*e.g.* Viefhaus *et al.*, 2013 ▸). Beamline P22 provides a bandwidth down to <50 meV at 5.977 keV using the Si(333) monochromator crystal. Convoluted with the ToF-resolution and thermal broadening this leads to a total resolution of 60 meV. Such resolutions of >10^5^ in HAXPES demand small entrance slits in hemispherical analyzers and good timing conditions in the ToF instrument. The 3D recording scheme sketched in Fig. 1(*a*) ▸ efficiently counteracts the reduced photon flux and dropping cross section.

### 2.2. High-energy *k*-space microscopy   

Alongside the high kinetic energy, the key feature of the new ToF momentum microscope is the increased diameter of the *k*-space images. This is achieved by a strongly enlarged electron optics (lens diameters 100 mm instead of previously 20–40 mm) and a DLD with 80 mm diameter (previously 40 mm). The result is a strongly enhanced *k*-field-of-view up to ∼25 Å^−1^ diameter, revealing many BZs in parallel. A particular challenge was the development of an objective lens that keeps the image aberrations small despite very large phase-space volumes. In addition, this lens allows imaging in the conventional acceleration mode (extractor at high positive voltage), at zero extractor field and in the space-charge suppression mode using a strong decelerating field in front of the sample surface. This aspect of space-charge correction and suppression is discussed by Schönhense *et al.* (2018*b* ▸) and Schönhense *et al.* (2015 ▸).

Momentum microscopy makes use of a basic concept of optics: in imaging systems the reciprocal image represents the distribution of the transversal momentum components (in mathematical language it is termed the Fourier image). Owing to *k*-conservation in the photoemission process, the reciprocal image formed in the backfocal plane of a cathode lens directly shows the transversal momentum distribution of the valence electrons inside the crystal. A compelling advantage is that this method yields the 2D (*k*_*x*_,*k*_*y*_)-distribution in a very large region without sample rotation or scanning deflector elements. Energy recording via ToF bears the advantage that many energy surfaces are acquired simultaneously. The present measurements employed the 40-bunch mode of PETRA III with a pulse period of 192 ns. In an experiment at MAX II, we used the full 100 MHz filling pattern, *i.e.* 10 ns pulse period, which was sufficient for core-level XPD studies. The DLD has a spatial resolution of ∼100 µm (resolving ∼0.5 megapixels) and its maximum integral count rate is 5 Mcounts s^−1^ at a time resolution of ∼150 ps.

Fig. 2(*a*) ▸ shows a schematic view and Figs. 2(*b*) and 2(*c*) ▸ the results of ray-tracing calculations for this microscope. The three lens groups are indicated in (*a*), Gaussian and reciprocal planes are denoted in (*b*). A special objective lens designed for minimum spherical aberration forms the first *k*-image in its back focal plane (BFP). The electron beam is transferred to the scattering energy of the spin filter (not shown here) by zoom optics 1 and to the desired drift energy in the imaging ToF spectrometer by zoom optics 2. Photoelectron momentum maps are taken simultaneously in an energy interval of width several electronvolts, limited by the chromatic aberration of the lenses. The simulations were based on the small footprint (∼20 µm) of the photon beam at P22, yielding a large depth of focus with ∼8 eV usable energy range. The large field of view in reciprocal space is imaged with negligible aberrations (the unavoidable field curvature is small). Time markers with 10 ns spacing (dots) reveal almost planar isochrones, an essential precondition for good energy resolution and no ‘crosstalk’ between longitudinal and transversal momentum components.

The example of Fig. 2(*b*) ▸ was calculated for an initial kinetic energy of 4 keV and an acceptance of 18 Å^−1^ diameter in *k*-space. The extractor field was switched on (1 kV mm^−1^), yielding the *k*-image of the near-normal-emission region. The example on the right of Fig. 2(*b*) ▸ is the Kikuchi pattern of a graphite single crystal, recorded on the C 1*s* core-level signal; the ToF spectrum is shown in (*a*). The new objective lens allows working without an acceleration field between sample and anode; in this mode the lens elements behind the first electrode focus the beam. The ray-tracing calculation of this mode is shown in Fig. 2(*c*) ▸, simulated for an initial energy of 8 keV. The example on the right of Fig. 2(*c*) ▸ is the off-normal C 1*s* Kikuchi pattern of a graphite single crystal, recorded for a sample tilt of 30° with respect to the optical axis of the microscope. Patching many such patterns would allow large angular ranges to be observed. Despite zero extractor field, *k*-fields as large as 16 Å^−1^ can be recorded without visible aberrations (corresponding to a full cone angle of 35° at 2.765 keV). For the same kinetic energy at the sample, a moderate extractor field of 1.5 kV mm^−1^ yields *k*-fields up to 22 Å^−1^, a larger field of 3 kV mm^−1^ yields *k*-fields up to 25 Å^−1^ on the 80 mm image detector and even larger fields for larger detectors. For a given signal strength this instrument yields an order-of-magnitude higher total intensity in comparison with the low-energy ToF *k*-microscope used in the soft X-ray range (Medjanik *et al.*, 2017 ▸). For full-field imaging microscopes the resolved number of *k*-points is limited by the detector, hence large *k*-fields like in Fig. 2 ▸ come at the expense of *k*-resolution. The *k*-resolution limit is defined by a typical value of 500 resolved *k*-points along the image diameter. This translates into a *k*-resolution of 0.03 Å^−1^ for large image fields like Fig. 2(*b*) ▸. Reduction of the *k*-field via the electron optics (zoom optics 2) improves the *k*-resolution accordingly.

### 2.3. ToF recording in the X-ray range   

The high photon energies pose a challenge to high-resolution ToF spectroscopy, especially because such instruments resemble high-pass filters. Hence, one might suspect that they are not suitable for core-level studies in general due to overlap of the signal with other core levels or the valence electrons. In addition, contributions of higher-order radiation from the beamline might complicate the situation further. In this section, we discuss the circumstances under which such undesired signals appear and possible solutions for their suppression. For the given conditions (*L* = 900 mm, dτ < 200 ps), the energy dispersion according to equation (2) is Δ*E*/meV = 0.235(*E*_d_/eV)^3/2^. We routinely achieve beam sizes of 15 µm × 15 µm. Owing to this small source area all measurements shown below were performed with open field aperture. The small source area acts as an effective ‘contrast aperture’ in *k*-imaging mode.

**Figure 3:**
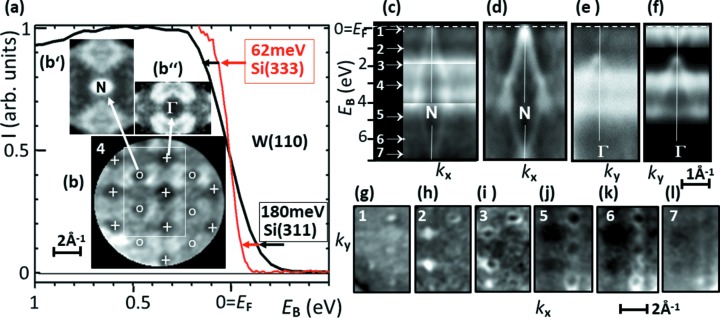
Energy-resolution measurements and valence-band mapping at *h*ν = 5.977 keV for the prototypical example W(110) at 30 K. (*a*) Fermi-edge cutoff spectra for the monochromator crystals Si(333) and Si(311). (*b*) Survey overview (*k*_*x*_–*k*_*y*_ section) and summed detail patterns of the regions around the high-symmetry points N (*b*′) and Γ (*b*′′). (*c*)–(*f*) *E*_B_-versus-*k* sections through high-symmetry points reveal a strongly improved signal-to-noise ratio in the sections through five summed N- (*d*) and Γ-point regions (*f*) in comparison to sections through a single N- (*c*) and Γ-point (*e*). (*g*)–(*l*) *k*_*x*_–**k**_*y*_ sections taken in the rectangular area marked in (*b*) at binding energies 1–7 as denoted in (*c*). The *k*-scales are quantified by the scale bars in (*b*), (*f*) and (*k*).

**Figure 4:**
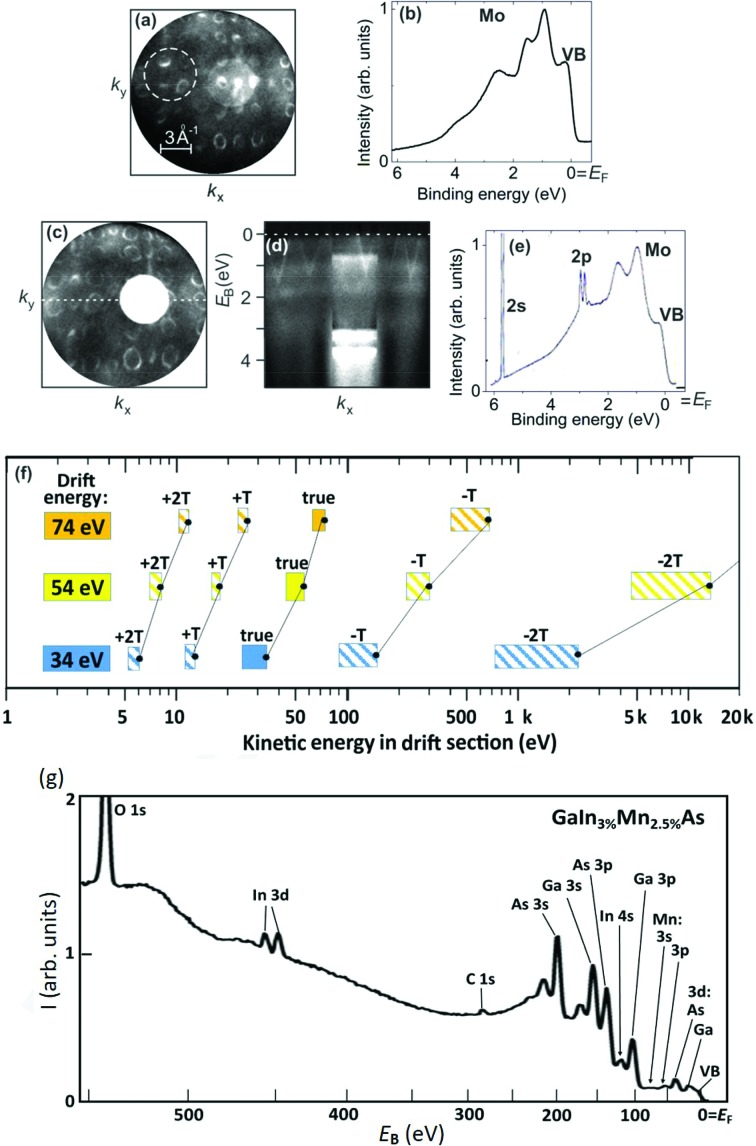
(*a*) (*k*_*x*_,*k*_*y*_) momentum distribution at *E*_F_ of valence-band photoelectrons from Mo(110) at *h*ν = 3.100 keV and (*b*) corresponding ToF spectrum taken from the dashed circle in (*a*). (*c*) (*k*_*x*_,*k*_*y*_) section of the same data set at a binding energy of 3.2 eV, where the 2*s*/2*p* core-level signals (bright circle) originating from a third-order admixture (*h*ν = 9.3 keV) incidentally coincide in time with the valence-band pattern. (*d*) (*k*_*x*_,*E*_B_) section along the dashed line in (*c*); (*e*) corresponding ToF spectrum taken in the white circle in (*c*). The *E*_B_ scale in (*e*) corresponds to the valence band (VB); the core-level electrons are much faster. (*f*) Temporal aliasing effect. Scheme of the energy intervals corresponding to the ‘true’ photoelectrons (10 eV intervals marked) and electrons which are slower or faster by one or two periods of the photon pulses (*T*) for drift energies of 34, 54 and 74 eV. The values correspond to the 80-bunch filling pattern of PETRA III (*T* = 96 ns; see text for details). Dots denote the Fermi edge of the true signal and the corresponding upper edges of the *nT*-bands. (*g*) Survey ToF spectrum (hence the non-linear energy scale) of a GaIn(3%)Mn(2.5%)As sample, recorded in static mode without scanning.

**Figure 5:**
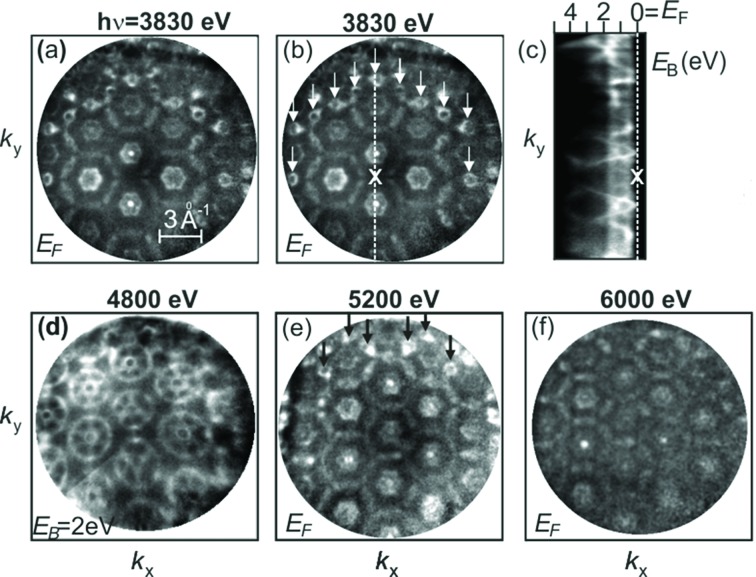
Hard X-ray valence-band mapping using the high-energy ToF momentum microscope. (*a*) Measured large-area *k*-space map at the Fermi energy for the *d*-bands of Re taken at *h*ν = 3.830 keV (*T* ≃ 30 K). The *k*_*x*_–*k*_*y*_ section shows a cut through the center BZ and the first and second ring of repeated BZs. (*b*) The same with marked small circular features, indicating the curvature of the final state energy isosphere in *k*-space. The top of the sphere is marked by a cross; the scale bar quantifies the momentum axes. (*c*) *E*_B_-versus-*k*_*y*_ cut along the dashed line in (*b*) showing the band dispersions. (*d*)–(*f*) *k*_*x*_–*k*_*y*_ sections at higher photon energies as given on top of the panels.

The binding energy (with respect to the Fermi energy *E*_F_) is determined as in classical photoemission (*E*_B_ = *h*ν − *E*_kin_ − Φ, where Φ is the work function) utilizing the relation *E*_kin_ = (1/2)*m*_e_(*L*/τ)^2^ [where *L*, τ and *m*_e_ are as in equation (2)]. For absolute ToF measurements, τ can be referenced to the photon signal itself (which is visible in the ToF spectra). In practical work, the energy scale is calibrated by a few measurements with slightly different sample bias yielding d*E*/dτ and observation of the Fermi edge providing the zero reference for *E*_B_. The DLD records all counting events in the selected *E* − *k* region confined by the isochrone surface at *E*_F_ and the selected diameter of the *k*-region of interest. For each counting event the three coordinates (*x*-position, *y*-position and arrival time τ) are registered and accumulated in the (*E*_B_,*k*_*x*_,*k*_*y*_)-voxels of a 3D data array. For visualization, either the full 3D object is displayed (for examples, see Chernov *et al.*, 2015 ▸) or sections can be cut along any plane (examples in Figs. 3, 4 and 5).

Fig. 3(*a*) ▸ quantifies the total energy resolution comprising photon bandwidth, ToF resolution and thermal broadening. The cut-off profiles of the Fermi edge of tungsten for monochromator crystals Si(333) and Si(311) at a photon energy of 5.977 keV reveal widths of 62 and 180 meV FWHM, respectively. For the Si(111) crystal we measured 450 meV at 4.0 keV. Deconvolution with the bandwidth of the X-ray beam [47 meV for Si(333)] and the small thermal broadening at 30 K reveals a resolution of the ToF-analyser of ∼40 meV. The measurements confirm an optical resolution of 1.3 × 10^5^, an excellent value for a HAXPES beamline. The analyser resolution of 1.5 × 10^5^ can compete with hemispherical analyzers and is far beyond all values reported for ToF-analyzers (for details see Section 2.4).

Figs. 3(*b*) ▸ show various sections through the measured *I*(*E*_B_,*k*_*x*_,*k*_*y*_) data array corresponding to the measurement in Fig. 3(*a*); this data array has been recorded in 20 min using the Si(311) crystal. Fig. 3(*b*) ▸ shows the full *k* field-of-view comprising many BZs with the Γ- and N-points marked by plus signs and circles, respectively. Recording multiple BZs allows for multiplexing of the observed band features, exploiting the periodicity of the bandstructure in *k*-space. This leads to an effective enhancement of the intensity (more precisely, to a reduction of the statistical noise) as visible in Figs. 3(*c*)–3(*f*) ▸. The sections through a single *N*-point (*c*) and Γ-point (*e*) look more diffuse than the sections through five summed *N*-points (*d*) and five Γ-points (*f*). In the section through *N* the outward-dispersing hole pocket (*d*) and through Γ the inward-dispersing electron octahedron close to *E*_F_, with adjacent local bandgap and hole-type occupied bands below at *E*_B_ > 1.25 eV (*f*), are clearly visible. Local details in the (*k*_*x*_,*k*_*y*_) sum patterns are shown on an enlarged scale as insets for *N* (*b*′) and Γ (*b*′′).

**Figure 6:**
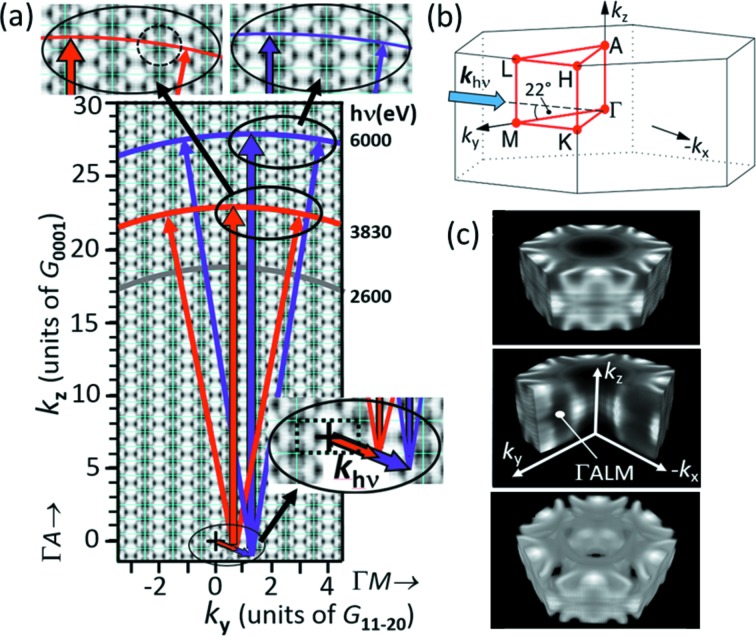
Quantitative analysis of the measured valence-band patterns of Fig. 5 ▸. (*a*) *k*-space scheme of direct transitions from the valence-band of Re to free-electron-like final states in the energy range used. The periodic background pattern is a measured Fermi-surface cut in this plane; the pattern is to scale with *k*_*z*_ and *k*_*y*_ quantified in multiples of the reciprocal lattice vectors ***G***_0001_ and 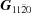 for Re. The dotted rectangle in the lower inset marks the first BZ. The three transitions shown lead to final-state energy isospheres crossing the 18th, 23rd and 28th repeated BZ along *k*_*z*_. ***k***_*h*ν_ is the photon momentum which lies in the drawing plane and leads to a displacement of the centers of the final-state spheres (shown in detail in the lower inset). (*b*) Brillouin zone with high-symmetry points and direction of photon impact. (*c*) Measured Fermi surface in different perspectives. The central panel denotes the cut leading to the background pattern in (*a*). Movies showing the measured momentum pattern at 3.830 keV as a function of binding energy, a *k*_*z*_-scan across the spectral density and a perspective view of the Fermi surface are shown in the supporting information.

In the rectangular area marked in (*b*) we took a sequence of (*k*_*x*_,*k*_*y*_)-sections at different binding energies 1–7 [as marked in (*c*)]. These cuts are shown in (*g*)–(*l*) and (*b*), the latter corresponding to binding energy 4. It is eye-catching that there are significant intensity differences between equivalent points in the sequence (*g*)–(*l*). These differences originate partly from the matrix element effect (different repeated BZs appear with different intensities) and partly from valence-band photoelectron diffraction (Schönhense *et al.*, 2018*a* ▸; Babenkov *et al.*, 2019 ▸). Such differences are largely compensated in the sum patterns, which is another advantage of BZ-multiplexing. For general aspects on background removal, see Babenkov *et al.* (2019 ▸). In addition, the curvature of the final-state energy isosphere (*cf*. Fig. 6) leads to a variation of *k*_*z*_ across the *k* field-of-view. For this reason, it is necessary to restrict the summing procedure to equivalent points not too far from the center of the observed momentum pattern, *i.e.* close to the maximum of the final-state sphere. Otherwise non-equivalent *k*_*z*_-sections of the *I*(*E*_B_,*k*_*x*_,*k*_*y*_,*k*_*z*_)-pattern in four-dimensional parameter space would mix.

In order to understand the problem of overlapping signals we recall some essentials of ToF recording. The *kinetic energy in the drift section* *E*_d_ is given by
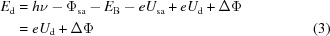
for the condition
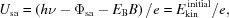
where Φ_sa_ is the work function of the sample and ΔΦ the work function difference between sample and drift tube; *U*_sa_ and *U*_d_ are the voltages at the sample and the drift tube. Then, only electrons in a band between *E*_F_ and *E*_B_ = *eU*_d_ are transmitted, with the topmost 8 eV being well focused. By using another lens as high-pass cut-off it is possible to restrict the energy interval further, as described by Tusche *et al.* (2016 ▸).

A ToF instrument also records faster photoelectrons, *e.g.* from higher orders (2*h*ν, 3*h*ν) of the monochromator/undulator. Photoelectrons released by higher-order photons are much faster and thus have a shorter ToF τ. If τ is shorter by one period of the exciting photon pulses (for the large storage ring PETRA III, 192 ns or 96 ns in the 40- or 80-bunch mode, respectively), higher-order signals appear in the same time interval as the photoelectrons from first order.

An example of an overlap of higher-order core-level signals with the valence-band (VB) is displayed in Figs. 4(*a*)–4(*e*) ▸. Panels (*a*) and (*b*) show the (*k*_*x*_,*k*_*y*_) momentum pattern at the Fermi energy and the corresponding ToF spectrum, taken in the dashed circle in (*a*), of a Mo(110)-sample excited by *h*ν = 3.100 keV. The second row shows the same data set in terms of a (*k*_*x*_,*k*_*y*_)-cut at a binding energy of 3.2 eV (*c*), where a strong core-level signal (bright circle) falls into the same time interval. The (*k*_*x*_,*E*_B_)-section (*d*) and the corresponding ToF spectrum (*e*) reveal that the Mo 2*s* and 2*p* core-level signals from a third-order admixture in the photon beam fall into the same time interval as the VB signal, although the 2*s* and 2*p* electrons are much faster. The binding energies are 2866 eV (2*s*) and 2625/2520 eV (2*p*_1/2,3/2_), *i.e.* for the third-order photon energy of 9.300 keV the kinetic energies of these undesired signals lie in the range of 6.4 to 6.8 keV.

The condition for this temporal coincidence is that the ToF τ of the undesired signal is shorter (or longer) than that of the true photoelectrons by one period *T* or a multiple *nT* of the exciting photon pulses. Since the clock of the DLD is started at each photon pulse, this undesired signal appears in the same time interval as the photoelectrons from first order. We have simulated the systematic behavior of this *temporal aliasing effect* (briefly *nT-effect*) for the present geometry and three drift energies typical for HAXPES. Fig. 4(*f*) ▸ shows the results for a large energy range from 5 eV to 20 keV and for the case of 80-bunch filling of PETRA III (pulse period *T* = 96 ns). The scheme shows the relevant cases between Δ*t* = −2*T* and +2*T*. For 40-bunch filling (*T* = 192 ns) the +*T* and −*T* intervals are missing, making the situation less complex.

Time-of-flight τ was determined using the model for the complete electron optics (Fig. 2 ▸) because the upstream part of the lens system also contributes, whereas equation (2) describes the drift section alone. The actual positions of the *nT*-bands depend on the drift energy, hence variation of *E*_d_ provides a simple means to shift a higher-order contribution out of the spectrum of interest. Another way is to change the photon energy, since the ToF of the true and higher-order signals respond differently. Being much faster, the higher-order electrons are essentially unfocused and pass the microscope column as a pencil beam, confined by the apertures in the ray path [white area in Fig. 4(*c*) ▸]. The different focusing conditions offer an alternative way to suppress the higher-order (−2*T*,−*T*) contributions: a combination of apertures and (weak) deflectors can be set such that electrons with the proper energy can pass but fast electrons are blocked. The same approach works for the electrons with lower energies (+2*T*, +*T*). However, since for these low drift energies the dispersion is very large, signals from slower electrons are usually strongly spread in time and are not visible as structures in the spectra. Temporal dispersion and energy resolution are strongly different for the true and ‘*nT*-shifted’ electrons, according to equation (2).

Fig. 4(*g*) ▸ shows a survey ToF spectrum of a GaInMnAs sample taken at a photon energy of 3.000 keV. The spectrum shows all outer core levels of the constituents, including the weak signals from indium (3%) and manganese (2.5%) as well as residual carbon and oxygen on the (unprepared) surface. For such a ToF survey spectrum (here 550 eV wide) taken in a single exposure with fixed setting of all lens voltages, the drift energy spans a large range.

**Figure 7:**
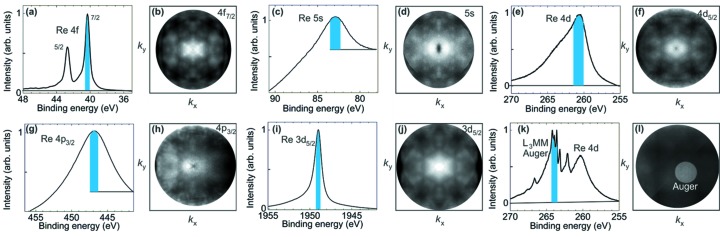
Core-level spectra and corresponding (*k*_*x*_,*k*_*y*_)-distributions of Re 4*f* (*a*,*b*), 5*s* (*c*,*d*), 4*d* (*e*,*f*), 4*p*_3/2_ (*g*,*h*) and 3*d*_5/2_ (*i*,*j*), recorded at *h*ν = 3.500 keV. (*k*,*l*) Group of *L*_3_ *MM* Auger lines excited by higher-order radiation, falling into the same time window as the Re 4*d* signal. Note that the binding energy scale in (*k*) refers to the 4*d* signal (at *h*ν = 3.750 keV); the Auger lines originate from third-order radiation (*h*ν = 11.250 keV). The blue bars denote the spectral ranges used for extracting the core-level diffractograms.

For the given spectrum the drift energy varies between 150 eV on the left-hand side and 700 eV at the Fermi edge. In turn, the energy resolution varies strongly from about 0.7 eV at the left-hand side to 3 eV at *E*_F_. The 3*s* signals are as strong as the 3*p* signals, reflecting that with increasing photon energy the partial cross sections of *s*-states drop much weaker than all other partial cross sections (Trzhaskovskaya & Yarzhemsky, 2018 ▸). Due to the fixed lens setting the transmission of the lens system increases with decreasing kinetic energy, *i.e.* from right to left in Fig. 3(*e*) ▸. Hence, the In 3*d* doublet appears relatively intense despite the small content of 2% and the oxygen 1*s* signal appears very strong although this is only a surface contamination. Detailed spectra of smaller energy ranges will be shown in Fig 7.

### 2.4. Comparison with existing types of spectrometers   

Full-field imaging of large 2D angular ranges is also exploited in the family of angular-resolving time-of-flight photoemission instruments and in various display-type analyzers. Here we discuss the similarities and differences of these approaches as compared with the present instrument, in particular concerning energy and angular/momentum resolution. The comparison includes also hemispherical analyzers that almost exclusively govern the field of HAXPES so far.

Three-dimensional photoemission recording has been implemented two decades ago in photoemission electron microscopy (PEEM) with ToF-analysis, by adding a low-energy drift space and an (*x*,*y*,*t*)-recording DLD (Oelsner *et al.*, 2001 ▸). This development was fueled by concepts for dynamic aberration correction (Schönhense & Spiecker, 2002 ▸). Angle-resolving ToF instruments as described by Ovsyannikov *et al.* (2013 ▸) and Berntsen *et al.* (2011 ▸) are based on electron-lens systems similar to those used in hemispherical analyzers, complemented by the same combination of drift section and DLD. ToF-ARPES instruments record a circular cone of emission angles and an energy interval of several eV simultaneously. This concept is similar to the *k*-microscope described in the present work. For photoelectrons with low kinetic energies and a moderate angular range (as accepted by the lens) the recording efficiency of ToF-ARPES instruments and the present microscope are identical. As long as both stay in the parameter window of 100% transmission, the total efficiency is just given by the detection sensitivity of the DLD. The recording schemes (polar coordinates in ToF-ARPES versus direct *k*-imaging) differ just by a simple transformation.

The *commercial ToF ARPES instruments* reach good values for the energy resolution at low energies. Previous work showed 4.7 meV at 10.2 eV laser excitation (Berntsen *et al.*, 2011 ▸), 5 meV at 7 eV laser excitation (Liang *et al.*, 2016 ▸), 50 meV for synchrotron excitation at 30 eV (Vollmer *et al.*, 2012 ▸), 20 meV at 230 eV (Kühn *et al.*, 2018 ▸), 30 meV at 90 eV and 800 meV at 810 eV (Kühn *et al.*, 2019 ▸). The high-energy ToF *k*-microscope is optimized for good resolution at very high kinetic energies; see results in Fig. 3(*a*) ▸. The measured total energy resolution of 62 meV FWHM at a kinetic energy of 5.972 keV can compete with the best HAXPES resolutions reported in the literature (Takata *et al.*, 2007 ▸; Torelli *et al.*, 2005 ▸; Suga & Sekiyama, 2014 ▸). Since the main contribution is given by the photon bandwidth of 47 meV for the Si(333) monochromator crystal, we estimate that the resolution of the ToF microscope itself is 40 meV. These values correspond to a total energy resolution (photons and ToF-analyzer) of 10^5^ and even 1.5 × 10^5^ for the analyzer alone. At low photon energies the ToF *k*-microscope has shown a resolution of 17 meV.

The second important parameter for electronic band mapping or photoelectron diffraction is the angular- or *k*-resolution and the accessible angular- or *k*-range. For the commercial ToF instruments, a maximum accessible *k*-field-of-view with radius 1.2 Å^−1^ is reported (Ovsyannikov *et al.*, 2013 ▸; Berntsen *et al.*, 2011 ▸). Previous work showed resolution values of Δ*k* < 0.02 Å^−1^ at 10.2 eV laser excitation (Berntsen *et al.*, 2011 ▸) and 0.05 Å^−1^ at 7 eV laser excitation (Liang *et al.*, 2016 ▸) and synchrotron excitation at 30 eV (Vollmer *et al.*, 2012 ▸). These values correspond to about 0.1° angular resolution, which is comparable with the resolution of the ToF *k*-microscope achieved in the soft X-ray range Δ*k* = 0.015 Å^−1^ (Medjanik *et al.*, 2017 ▸). At low energy (He I, 21.2 eV) a resolution of Δ*k* = 0.005 Å^−1^ has been achieved with the same imaging optics as the ToF *k*-microscope (Tusche *et al.*, 2015 ▸). In the hard X-ray range the present instrument achieved Δ*k* = 0.025 Å^−1^, equivalent to an angular resolution of 0.034° at 7 keV (Fedchenko *et al.*, 2019 ▸). At the maximum *k*-field-of-view (radius 12.5 Å^−1^) the resolution is diminished to ∼0.06 Å^−1^, because it is limited by 500 resolved image points along the image diameter.

ToF-ARPES spectrometers work for initial energies in the soft X-ray range with a resolution of ∼10^3^ (Kühn *et al.*, 2018 ▸). Owing to the high resolution at energies up to ∼100 eV, this family of instruments is very well suited for the analysis of small-momentum objects in *k*-space at rather low kinetic energies. Scanning of a full Brillouin zone would involve sample rotation.

*Momentum microscopes* take *k*-space images directly on a linear *k*-scale, without necessary transformation. The complete separation of imaging column and long field-free ToF section avoids crosstalk of longitudinal and transversal momentum components [see, for example, Fig. 2 of Kühn *et al.* (2018 ▸)]. In other words, the isochrone planes are practically planar [dots on the trajectories in Figs. 2(*b*) and 2(*c*) ▸]. Imaging of the intermediate real-space image (PEEM mode) allows inspection of the sample surface. Moreover, it can be used as a precise monitor for the position of the photon footprint. For small photon spots like at beamline P22 the adjustable field aperture for selection of the region-of-interest on the sample is not needed.

An alternative way of accessing large solid-angle intervals has been implemented in the *display-type analyzers* by using a combination of grids. As early as 1980, Eastman *et al.* developed the first instrument of this kind with a filter stage consisting of an ellipsoidal electron mirror as low-pass and spherical grid as high-pass (Eastman *et al.*, 1980 ▸). Later, Daimon *et al.* and other groups published several improved design studies (Daimon, 1988 ▸; Daimon *et al.*, 1995 ▸; Matsuda *et al.*, 2014 ▸), in particular a display-type spherical-mirror analyzer that can obtain atomic stereoscopic images (Daimon, 2001 ▸). Imaging almost the full half space simultaneously at a given energy, these instruments achieved a wealth of good results (Guo *et al.*, 2006 ▸; Daimon *et al.*, 1998 ▸; Kato *et al.*, 2007 ▸; Matsui *et al.*, 2014 ▸, 2018 ▸). The micro-lens action of the grid meshes sets a principal limit to the achievable energy and angle resolution (Matsuda *et al.*, 2014 ▸); this restriction can be reduced with ultrafine-mesh grids. These instruments are window-type spectrometers, *i.e.* they acquire 2D images and the spectral information is retrieved by scanning the grid potentials.

So far, HAXPES was the domain of *hemispherical analyzers*. Energy resolutions in the 100 meV range are routinely achieved at optimized beamlines, values of 55 meV at 7.9 keV (Takata *et al.*, 2007 ▸) and 71 meV at 5.9 keV (Torelli *et al.*, 2005 ▸) have been reported for measurements at the Au Fermi edge, comparable with our result for the W Fermi edge in Fig. 3(*a*) ▸. Hemispherical analyzers also dominate the field of photoelectron diffraction. One main emphasis in XPD was to observe the pronounced forward scattering along atom rows in off-normal directions (Fadley, 2010 ▸; Fadley *et al.*, 1995 ▸), driving the trend to observe a maximum polar angular range. Large ranges of typically 0–60° can be scanned by varying the polar angle and rotating the sample about its surface normal (*e.g.* Fadley, 2010 ▸; Woodruff, 2010 ▸; Osterwalder, 2011 ▸; Fasal *et al.*, 1995 ▸; Shamout *et al.*, 2018 ▸). Typical angular resolutions in both angular-scanning and display-type recording modes are ∼1°. In a few cases higher resolutions (<1° FWHM) have been reached (Osterwalder *et al.*, 1994 ▸; Katayama *et al.*, 1999 ▸; Broekman *et al.*, 2005 ▸). This approach works well in the hard X-ray range as demonstrated even with a laboratory X-ray source (Kobayashi *et al.*, 2013 ▸). In the present instrument, the angular range observable without scanning is given by the maximum *k*-field-of-view (∼25 Å^−1^). The corresponding simultaneously imaged angular range varies from 30° half cone angle at 2.5 keV to 17° at 7 keV; details on this mode are discussed by Fedchenko *et al.* (2019 ▸). Beyond that range sample rotation is required, which demands zero extractor field [see example in Fig. 2(*c*) ▸]. In the high-angular-resolution mode (Δθ ≃ 0.1°), hemispherical analysers typically record a range of emission angles of ±7° (along one direction in *k*-space).

This comparison would not be complete without considering the time structure required for the ToF methods. Modern high-performance photon sources (storage rings, FELs, laser-based HHG sources, low-energy laser sources) are all characterized by a time-structure with pulse widths sufficiently small for ToF operation. The time resolution is limited not by the source but by the detector. The present generation of DLDs have time resolutions of ∼150 ps which will soon be improved by a factor of two in a new generation of DLDs with different recording architecture [http://www.surface-concept.de]. An obstacle is posed by the repetition rate, because the desired spectrum has to be ‘squeezed’ into the time interval between adjacent photon pulses. Aiming at 1000 resolvable points in a full energy spectrum (more precisely, 1000 resolvable time slices), intervals of 200 ns or more are fine. This translates into pulse rates of 5 MHz or less. The data shown below have been recorded using the 40-bunch mode of PETRA III (5 MHz); this criterion is also fulfilled at the free-electron laser FLASH [1 MHz, where ToF *k*-microscopy has been successfully implemented (Kutnyakhov *et al.*, 2019 ▸)], the European XFEL (where such a microscope will be installed at SASE3), and further upcoming FEL sources like LCLS II. The most common 500 MHz filling pattern of most storage rings is not directly suitable for ToF-recording. However, the effective pulse rate can be reduced by a synchronized mechanical chopper, selecting, for example, the camshaft pulse in the filling pattern (Kühn *et al.*, 2018 ▸). Another approach is based on the generation of ‘transverse-resonance island buckets’ on a second stable orbit in the ring that can be filled with a lower bunch number. The latter approach was implemented at BESSY II in a test beam time (Goslawski *et al.*, 2019 ▸) and the second orbit filled by a single bunch could be used in the ToF *k*-microscope parallel to standard multi-bunch operation of the main orbit.

## 3. Applications   

### 3.1. Mapping of bulk valence-bands   

One of the key questions addressed in the first pilot experiment was whether mapping of the electronic structure using *k*-microscopy works in the hard X-ray range. Although valence-band features had been observed at high X-ray energies (Claesson *et al.*, 2004 ▸; Kamakura *et al.*, 2004 ▸; Sekiyama *et al.*, 2004 ▸; Plucinski *et al.*, 2008 ▸; Papp *et al.*, 2011 ▸; Gray *et al.*, 2011 ▸, 2012 ▸; Keqi *et al.*, 2018 ▸; Nemšák *et al.*, 2018 ▸; Sato *et al.*, 2018 ▸; Kobayashi *et al.*, 2014 ▸), it seemed that they are strongly masked by quasi-elastic phonon scattering. The background of such electrons exhibits a characteristic spectral distribution given by the ‘matrix-element weighted density of states’ (Osterwalder *et al.*, 1990 ▸, 1996 ▸; Herman *et al.*, 1992 ▸). The point where density-of-states information dominates over direct transitions (termed XPS limit) was explored theoretically (Braun *et al.*, 2013 ▸; Nicolaï & Minár, 2018 ▸). Moreover, it was not clear whether the *k*_*z*_-resolution stays sufficiently high in the several-keV range. This is a necessary precondition for precise mapping of bulk bands. The transfer of photon momentum ***k***_*h*v_ to the photoelectron leads to a substantial shift of the *k*-pattern. The interplay of this shift with diffraction of the photoelectrons from the valence-band is still in an early stage of understanding (Schönhense *et al.*, 2018*a* ▸). Last but not least, typical count rates of conventional HAXPES spectrometers seemed prohibitively low for extended 4D mappings. This would require 3D data arrays with good counting statistics taken at many different photon energies in order to vary *k*_*z*_. In this section we will show that mapping of the 4D spectral function ρ(*E*_B_,***k***) in a tomography-like manner as introduced in the soft X-ray range (Medjanik *et al.*, 2017 ▸) still works for hard X-rays, if the momentum conservation including the photon momentum is properly accounted for.

Fig. 5 ▸ shows results for rhenium taken at photon energies up to 6 keV. Fig. 5(*a*) ▸ shows a *k*_*x*_–*k*_*y*_ cut (at *E*_F_) through the measured data array for the Re(0001) sample at *h*ν = 3.830 keV; all patterns shown were measured at low sample temperature (*T* ≃ 30 K). Each of the sixfold rings represents one BZ; we see one in the center, a first ring with six and a second ring with 12 adjacent BZs, making up 19 BZs observed in parallel. Accumulation time was ∼15 min at a total count rate of ∼10^6^ counts s^−1^. A slight image-field curvature (due to the large field of view) and Lorentzian deformation due to a weak but significant space-charge shift was visible. The Coulomb interaction of a photoelectron with the charge cloud of slow secondary electrons leads to an anisotropic acceleration of the photoelectrons with a Lorentzian profile. This deformation of the energy distribution in *E*_kin_ versus *k* representation can be very strong in the soft X-ray range [with shifts up to 10 eV (Schönhense *et al.*, 2018*b* ▸)]. In the hard-X-ray range the effect is an order of magnitude weaker, probably due to the lower cross section and high speed of the photoelectrons, leaving the cloud of low-energy (true) secondary electrons very rapidly. The deformation can be corrected using the algorithms described by Schönhense *et al.* (2018*b* ▸).

The background due to phonon scattering was removed by the procedure described by Gray *et al.* (2011 ▸), the lateral intensity modulations due to valence-band photoelectron diffraction were removed as described by Babenkov *et al.* (2019 ▸); this type of valence band diffraction will be discussed in Section 3.3.

It is eye-catching that in Fig. 5(*a*) ▸ and all other *k*_*x*_–*k*_*y*_ distributions different BZs look differently and the patterns do not exhibit the full sixfold symmetry which we would expect for Re(0001). Instead, a twofold symmetry modulates the pattern of sixfold rings. The center of this apparent twofold symmetry is marked by a cross in Fig. 5(*b*) ▸. The reason for this – at first sight puzzling – deviation from the expected symmetry becomes clear when we take into consideration that the final states lie on a curved energy isosphere [see details on top of Fig. 6(*a*) ▸]. Due to the increased field of view of the present instrument, the curvature of the intersection contour of the sphere and the periodic *k*-space pattern becomes substantial. In the previous experiments in the soft X-ray range, focusing on one BZ, this effect was just a small correction. The ‘symmetry point’ marked by X in Fig. 5(*b*) ▸ corresponds to the top of the sphere and, with increasing distance from this point, the *k*_*z*_-value is reduced. In turn, the cut runs through different features of the 3D Fermi surface. As a guide to the eye, we marked the small circles in the Fermi-surface cut with arrows in (*b*). These circles appear when the sphere intersects a small Fermi-surface pocket located about midway between the ΓMK and ALH symmetry planes [*cf*. the BZ in Fig. 6(*b*) ▸]. In the observed patterns, these circles lie on a ring because these points have the same distance from the top point X; see the dashed circle in the upper left inset of Fig. 6(*a*) ▸.

The curvature of the final-state sphere is also visible in the *E*_B_-versus-*k*_*y*_ cut, Fig. 5(*c*) ▸. The centers of the different BZs are denoted by Γ (keeping in mind that this is not exactly the bulk Γ-point). We see that the downward-dispersing band only reaches the Fermi energy in the two points next to ‘X’. The 4D nature of the spectral density function ρ(E_B_,***k***) along with the *a priori* curved final-state isosurface and the broken symmetry by the action of the photon momentum lead to a complex behavior. Figs. 5(*d*)–5(*f*) ▸ show maps measured at higher photon energies. Fig. 5(*d*) ▸ corresponds to a binding energy of 2 eV, whereas all other panels in Fig. 5 ▸ show cuts at the Fermi energy. For Figs. 5(*a*), 5(*e*) and 5(*f*) ▸ we have chosen energies that lead to equivalent ‘planes’ in different repeated BZs. Since the curvature of the sphere is reduced with increasing *h*ν, the small circles are shifted outwards [compare arrows in Figs. 5(*b*) and Figs. 5(*e*) ▸], thus confirming the transition scheme proposed in Fig. 6 ▸.

The experimental results of Fig. 5 ▸ are confirmed by the quantitative momentum-space scheme in Fig. 6(*a*) ▸. In a *k*-space description, the photo-transition into a free-electron-like final state leads to an energy isosphere centered at ***k*** = (0,0,0), reflecting energy-conservation analogously to the Ewald sphere in diffraction. The scheme in Fig. 6(*a*) ▸ is to scale, with *k*_*z*_ and *k*_*y*_ being quantified by the reciprocal lattice vectors along the directions Γ*A* and Γ*M* for the hexagonally close-packed (h.c.p.) metal Re, ***G***_0001_ (1.410 Å^−1^) and 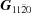 (2.276 Å^−1^), respectively. Plotting the transitions for three selected photon energies in the accessible range reveals final-state isospheres crossing the region between the 18th and 28th repeated BZs along *k*_*z*_. The dashed rectangle in the lower inset in Fig. 6(*a*) ▸ denotes the ΓALM-cut of the first BZ, shown in Fig. 6(*b*) ▸. The background pattern is the spectral density function as will be discussed below. The transfer of photon momentum ***k***_*h*ν_ to the photoelectron causes a strong displacement of the center of the final-state energy isospheres from the origin ***k*** = (0,0,0) (marked by +) by as much as 3.04 Å^−1^ at 6 keV. Due to the impact angle of 22° from the surface, the main shift (|***k***_*h*ν_|cos22° = 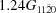) acts in the lateral direction and a smaller shift (|***k***_*h*ν_|sin22° = 0.81*G*_0001_) in the perpendicular direction. ***k***_*h*ν_ is proportional to *h*ν, whereas the photoelectron momentum *k*_f_ increases with the root of the energy (the equation denotes the final-state momentum *k*_f_ inside of the solid),
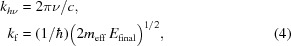
with 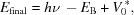
where binding energy *E*_B_ and inner potential 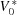 are referenced to the Fermi energy. In the hard X-ray range, the final-state effective mass *m*_eff_ is identical to the free-electron mass (Gray *et al.*, 2011 ▸). Growing linearly with *h*ν, the photon-momentum-induced shift becomes very substantial at high energies. For Re at 6 keV, it shifts the center of the final-state sphere transversally by more than a full BZ [see lower inset in Fig. 6(*a*) ▸]. This rigid shift of the final-state sphere with radius *k*_f_ leads to a significant dissymmetry in the observed photoemission patterns as illustrated by the top insets in Fig. 6(*a*) ▸. In *k*-microscope images, this rigid shift by ***k***_*h*ν_ is directly observable as discussed by Medjanik *et al.* (2017 ▸).

Note that the top of the sphere is not located on the Γ–A line crossing the center of the BZ but is shifted according to the size and direction of the photon momentum vector. The role of ***k***_*h*ν_ in HARPES is not at all trivial and influences the observed patterns substantially. The final-state sphere is just rigidly shifted in 3D *k*-space. However, concerning the observed pattern, this shift shows up in a complex way which can only be understood in a full 3D *k*-space transition model. Fig. 6(*a*) ▸ shows the plane which contains ***k***_*h*ν_, hence perpendicular to this plane there is no photon-momentum component. This causes the apparent two-fold symmetry. Experimentally, the direction perpendicular to the surface can be identified by the profile of the space-charge induced Lorentzian energy shift (Schönhense *et al.*, 2018*b* ▸) and by the center of the Kikuchi-type diffraction pattern (*cf*. Section 3.3). The point with initial states with *k* = 0 is displaced from the perpendicular direction by |***k***_*h*ν_|cos22° as discussed above. Energy conservation (leading to a final-state energy isosphere) acting independent of momentum conservation including ***k***_*h*ν_ (leading to a shift of this sphere in *k*-space) gives rise to striking dissymmetries in the photoemission patterns. The scenario differs fundamentally from the conventional view of transitions from an initial band state into a free-electron-like parabola.

Fig. 6(*c*) ▸ shows the measured Fermi surface of Re, *i.e.* the spectral-density function ρ(*E*_F_,***k***) (actually the ‘removal part’ that is accessible by photoemission) at the Fermi energy. This object in 4D energy-momentum parameter space is determined as follows. First, a tomographic-like sectioning of a unit cell in *k*-space is performed by measurement of 3D *I*(*E*_B_,*k*_*x*_,*k*_*y*_) data arrays at ∼20 different photon energies, crossing a full BZ. Next, individual energy isosurfaces at *E*_F_ are obtained from these arrays. Finally, these arrays are concatenated to form the complete Fermi surface in 3D *k*-space; for more details on the procedure, see Medjanik *et al.* (2017 ▸). The measurements leading to Fig. 6(*c*) ▸ have been made in the soft X-ray range (at PETRA beamline P04), with its intrinsically higher energy resolution. This experimental Fermi surface was the basis for the background pattern in Fig. 6(*a*) ▸, which is a cut at *k*_*x*_ = 0 (*i.e.* the ΓALM-plane) of the periodically repeated Fermi surface. Note that in Fig. 6(*a*) ▸ the contrast is inverted for clarity (dark denotes high spectral density).

The curvature of the final-state sphere intersecting the periodic pattern of BZs offers a fascinating possibility for future electronic structure analysis. A large *k*-field-of-view on the curved isosphere intersects several BZs with continuously varying *k*_*z*_ value. In Fig. 5(*a*) ▸ *k*_*z*_ spans already half of the Γ–A distance of the BZ. The highest point is marked by X, the BZs above and below the X show an incomplete ring with three bars only and an inner hexagon with a bright spot in the center. The adjacent BZs left and right from X show complete rings with six bars and the spot in the inner hexagon is much weaker. The next nearest BZs in the upper part of the field of view show the small circles (arrows) originating from a Fermi-surface pocket located about midway between the ΓMK and ALH planes. These variations originate from the curved isosphere and thus are equivalent to a *k*_*z*_-scan varying the photon energy. The access to different *k*_*z*_-levels exploiting the radial coordinate in the *k*-images allows, in principle, mapping of the spectral function via measurements at few photon energies or even just one photon energy (if the imaged *k*-field is large enough and/or the size of the BZ along *k*_*z*_ is small). In this context the possibility to work in the zero-extractor mode [Fig. 2(*c*) ▸] and tilt the sample gives access to *k*-regions far away from the *k* = 0 point.

### 3.2. ToF core-level spectroscopy   

Core-level spectroscopy is a fingerprint method for the element composition and the chemical state of elements in a compound. The real-space imaging mode allows to precisely locate the analyzed area on the sample. Microspectroscopy is possible with spatial resolution better than the photon footprint by using the size-selectable *field aperture* in the intermediate real image plane [Fig. 2(*b*) ▸]. Momentum images of photoelectrons from core levels represent XPD patterns. They carry information on the geometrical structure, complementary to the electronic structure information of the valence-band patterns. Owing to their large cross section in comparison with the valence band, the *k*-distribution of most core levels is recorded in extremely short time: in the present experiments, a few minutes for a pattern with good statistics. Here we discuss the spectroscopic aspect and in Section 3.3 the diffraction aspect.

Fig. 7 ▸ shows a collection of core-level spectra for rhenium along with the corresponding *k*-distributions. Technically, the change from valence-band mapping to a core level only requires setting the sample bias *U*_sa_ to the proper value, according to equation (3). Only for very large changes of the kinetic energy might a slight refocusing of the objective lens be necessary. We recorded spectra and *k*-patterns of the Re 4*f* doublet [(*a*,*b*), *E*_B_ = 40.5/42.9 eV], the 5*s* line [(*c*,*d*), *E*_B_ = 83 eV], the 4*d*_5/2_ line [(*e*,*f*), *E*_B_ = 261 eV], the 4*p*_3/2_ line [(*g*,*h*), *E*_B_ = 447 eV] and the 3*d*_5/2_ line [(*i*,*j*), *E*_B_ = 1883 eV]. All core-level spectra and diffractograms show excellent signal-to-noise ratios after a few minutes of acquisition time. For core levels with very large cross sections, attenuators had to be inserted in the photon beam reducing the count rate to ∼2 Mcounts s^−1^ to avoid multi-hit events originating from the Poisson statistics. Such events cannot be properly analyzed by the DLD and thus increase the background level (the photon pulse rate was 5 MHz). Using denser filling patterns of the storage ring, this problem would be eliminated and the 8 MHz count-rate capability of the detector could be fully exploited. The intensity scales in Fig. 7 ▸ [except (*c*) and (*g*)] show the true zero, *i.e.* despite the high-pass characteristic the background levels are not larger than in a hemispherical analyser. The spin–orbit splitting of Re 4*d* (13 eV), 4*p* (72 eV) and 3*d* (66 eV) is too large to fit into the selected energy interval at the given settings, hence only the line with the higher total angular momentum is recorded. Much larger energy ranges can be recorded at higher drift energies, *cf*. Fig. 4(*g*) ▸. All diffractograms are very pronounced and rich in detail, as will be discussed in Section 3.3.

The fortuitous coincidence of the investigated spectrum with a signal originating from higher order of the monochromator [explained in Fig. 4(*f*) ▸] can also happen in core-level spectra. An example is shown in Fig. 7(*k*) ▸, where a group of *L*_3_ *MM* Auger lines falls onto the low-energy wing of the Re 4*d* line. The higher energy of the Auger signal is evident from the small circular region in which this signal is present, *i.e.* these electrons are much faster than the true 4*d* core-level electrons. Spectrum (*k*) was taken in the area of the bright circle in (*l*). It is straightforward to estimate the energy of this Auger signal. At the given photon energy of 3.750 keV, the kinetic energy of the 4*d*_5/2_ electrons is 3.484 keV (assuming a work function of 5 eV). These electrons are then retarded to 60 eV drift energy in the ToF section (*i.e.* their energy is reduced by 3424 eV). The Auger electrons arrived one period of the photon pulses (*i.e.* 192 ns) earlier. The fast Auger electrons from photon pulse #*n* coincide in time with the 4*d* electrons from photon pulse #*n* − 1. Using the simulation program with the proper lens settings, we arrive at an initial kinetic energy of the Auger electrons of ∼6000 eV, following photoemission from an inner shell by third-order photons with *h*ν = 11.250 keV. Consulting Auger tables we arrive at the *L*_3_ *MM* cascade with kinetic energies of ∼5970 eV (∼6219 eV) for the *L*_3_ *M*_2_ *M*_5_ (*L*_3_ *M*_3_ *M*_4_) transitions. Note that the energy scale of the Auger signal is much wider due to the much higher drift energy of these electrons. Changing the photon energy to 3.500 keV shifts the undesired Auger signal out of the recorded time window [Figs. 7(*e*) and 7(*f*) ▸].

### 3.3. Core-level photoelectron diffraction   

There is a huge amount of XPD data in the literature. Previously, XPD results have been obtained using two different techniques. (i) Conventional XPS analyzers in combination with two-axes sample rotation allow scanning large polar angular ranges (typically 0–60°) and full azimuthal scans. This method was pioneered by Fadley and co-workers (Fadley, 1987 ▸, 1992 ▸, 2012 ▸; Winkelmann *et al.*, 2008 ▸), and later used by several other groups (*e.g.* Woodruff, 2010 ▸; Osterwalder, 2011 ▸; Fasel *et al.*, 1995 ▸; Shamout *et al.*, 2018 ▸). (ii) Display-type analyzers record 2D patterns in parallel in a large polar angular range of 0–75° without rotation of the sample (see references in Section 2.4).

**Figure 8:**
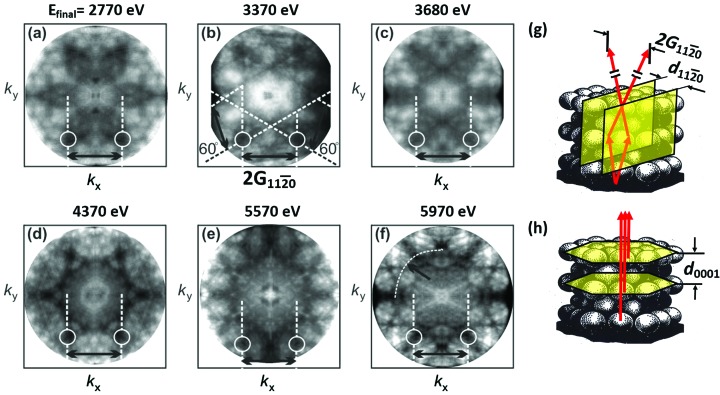
Sequence of photoelectron diffraction patterns of the rhenium 4*f*_7/2_ core-level signal (*E*_B_ = 40.5 eV) at selected photon energies between 2.8 and 6 keV, corresponding to the final-state energies given in panels (*a*)–(*f*) (*E*_final_ = *h*ν − *E*_B_ + 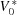). The patterns have been symmetrized; the contrast has been optimized in order to emphasize fine features. Dashed lines denote the principal Kikuchi bands (width 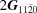 = 4.552 Å^−1^) originating from Bragg reflection at lattice planes perpendicular to the surface. In (*b*) the sixfold symmetry of the bands is indicated. White circles mark identical crossing points of dark Kikuchi lines, providing a convenient ‘ruler’ in *k*-space. (*g*,*h*) Model of the h.c.p. lattice of Re. Lattice planes with distance 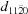 cause the Kikuchi bands (*g*); planes with distance *d*_0001_ cause constructive diffraction in the forward direction normal to the surface (*h*).

The full-field acquisition scheme of a time-of-flight *k*-microscope is ideal for rapid recording of XPD patterns without changing the sample geometry. However, the angular range is much smaller than in conventional experiments. A sequence of photoelectron diffraction patterns for the Re 4*f*_7/2_ core-level signal taken between *h*ν = 2.8 and 6 keV is shown in Fig. 8 ▸. Since the quantity relevant for photoelectron diffraction is the final-state energy (kinetic energy of the photoelectron inside the sample) as defined in equation (4), the panels state *E*_final_ instead of the photon energy. These small-angle diffractograms show a surprisingly rich structure, which varies strongly with photon energy. Translated into real-space polar coordinates, the diameters of the *k*-images correspond to angular ranges between 0–15° at 2.770 keV and 0–11° at 5.970 keV. Some fine features have widths <0.1 Å^−1^, corresponding to <0.1°; the angular resolution of the instrument is 0.034°.

Core-level signals are selected by setting the proper sample bias, such that the binding-energy region of interest is accumulated [*cf*. equation (3)]. In other words, the kinetic energy *E*_kin_ of the core-level signal is shifted exactly to the point of best focusing of the electron optics. As discussed in Section 2.3, faster electrons originating from higher-lying core levels, from the valence band or from admixture of higher orders in the photon beam, usually appear in different time slices or can be shifted out of the spectrum. Electrons slower than *E*_kin_ − *eU*_d_ are cut off by the high-pass action of the drift tube. At the given conditions [Si(111) monochromator crystal, 450 meV bandwidth, 40-bunch filling pattern], diffractograms like those in Fig. 8 ▸ were obtained in ∼10 min. In the future, multi-line DLDs (http://www.surface-concept.de) with highly parallelized recording architecture (and high multi-hit capability) will allow for much higher total count rates. The space charge problem is less serious when recording XPD, because quasi-monoenergetic core-level patterns can be extracted much easier than valence-band patterns, even when Coulomb repulsion in the beam leads to a Lorentzian deformation of the raw data [*cf*. Schönhense *et al.* (2018*b* ▸) for details].

In order to observe a larger radius, the symmetry centers of the patterns in Fig. 8 ▸ were shifted off the image center, and the measured distributions were symmetrized. The electric vector of the incoming X-ray beam is oriented 22° off the surface normal. The as-measured data show that this symmetry breaking has only a minor effect on the observed patterns. The initial directional characteristic is ‘washed out’ by the multiple-scattering processes, hence the diffractograms reflect essentially the sixfold symmetry of the Re (0001) crystal. The same argument holds for the action of the photon momentum, which was crucial in the direct transitions from the valence-bands. Here the fingerprint of the photon momentum is absent and the center of the XPD pattern coincides with the symmetry axis of the crystal, here the [0001] direction.

At first sight, the patterns in Fig. 8 ▸ do not exhibit systematic features that allow for an analysis. Instead, all patterns look strongly different and seem to have nothing in common except for the sixfold symmetry. In large angular ranges, XPD patterns are dominated by strong forward scattering along atom rows (Fadley, 1987 ▸, 1992 ▸, 2012 ▸; Winkelmann *et al.*, 2008 ▸), allowing for an analysis in real-space (polar) coordinates. Luckily, some of our small-angle diffractogram, Figs. 8(*b*), 8(*c*) and 8(*f*) ▸, exhibit patterns with straight *Kikuchi lines*, which allow for an orientation in *k*-space. Kikuchi lines are conveniently explained in terms of Bragg reflection at lattice planes (Kikuchi, 1928 ▸). In the small angular range close to normal emission, we expect to see a sixfold-symmetric pattern of Kikuchi bands, originating from Bragg reflection at sets of lattice planes perpendicular to the surface. Fig. 8(*g*) ▸ shows a model of the Re lattice with marked lattice planes with spacing 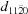. In a momentum image this set of planes leads to a Kikuchi band of width 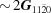 (4.552 Å^−1^), confined by dark lines on both sides. Owing to the small photoelectron wavelength, the Bragg angle is very small. At *E*_final_ = 5.970 keV we have |***k***_final_| = 38.4 Å^−1^ [taking into account the photon momentum contribution according to equation (4)] and θ_Bragg_ = 3.4°, so that the Bragg-scattered electrons appear in our field of view. Note that the lower part of Fig. 8(*g*) ▸ is a real-space model, whereas the upper part is drawn in *k*-space coordinates. The clearest signature of Kikuchi bands crossing under ±60° is visible at *E*_final_ = 3.370 keV as indicated by the dashed lines in the lower part of Fig. 8(*b*) ▸. Particularly prominent points in all patterns are the crossing points of dark lines confining the Kikuchi bands. These points can be identified in all diffractograms (*a*–*f*); two of these dark crossing points are marked by circles in all panels of Fig. 8 ▸. We conclude that, although patterns (*a*–*f*) look quite different, they all carry the signature of the sixfold Kikuchi band, originating from Bragg reflection at the 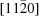 lattice planes.

In addition to the Kikuchi pattern, diffractogram Fig. 8(*b*) ▸ shows a significant intensity enhancement in the center region. This originates from diffraction of the photoelectron wave at lattice planes parallel to the surface as sketched in (*h*). Actually, this constitutes the simplest case of quasi-1D diffraction and is equivalent to constructive forward scattering along atom rows perpendicular to the surface. At 3.370 keV, the final-state momentum along *k*_*z*_, corrected by the photon momentum according to equation (4), is ***k***_f_ = 20.5***G***_0001_. This condition corresponds to the Bragg condition for constructive interference in normal emission [for details, see equation (6) of Schönhense *et al.* (2018*a* ▸)]. The enhanced intensity in the center of (*b*) is caused by forward scattering.

The 5.970 keV pattern, Fig. 8(*f*) ▸, shows pronounced dark straight lines with sixfold symmetry but different from Fig. 8(*b*) ▸. These straight lines could be *Kikuchi lines*, originating from Bragg reflection at lattice planes tilted against the surface normal (Fedchenko *et al.*, 2019 ▸). Most features, *e.g.* the leaf-shaped dark regions in (*a*), the filigree-like central regions in (*d*)–(*f*), with small spots and circles do not directly fit to a Kikuchi-band pattern. This rich structure appears because the Kikuchi patterns of heavy materials like rhenium are strongly distorted due to the large scattering factor of high-*Z* elements. Distortions are strongest in regions where several Kikuchi bands cross each other, like the central region in Figs. 8(*a*)–8(*f*) ▸. Since the kinetic energy varies strongly, there is a slight systematic change in the field of view due to the imaging characteristics of the objective lens. The energy dependence of this effect is precisely known and the images have been calibrated to equal *k*-scales.

It is eye-catching that the sharpness of the features in Fig. 8 ▸ increases with energy. When increasing the final-state energy from 2.77 to 5.97 keV [sequence (*a*)–(*f*)], two trends are important: the photoelectron wavelength drops from 23.2 pm to 15.8 pm and the inelastic mean free path λ_IMFP_ increases from ∼3 nm to ∼5.4 nm. Photoelectron diffraction can be described in a real-space cluster model (Trehan *et al.*, 1987 ▸; Krüger *et al.*, 2011 ▸), which is equivalent to the Bloch-wave model (Winkelmann *et al.*, 2008 ▸) if both are fully converged. Significant diffraction contributions can be expected until the amplitudes of the scattered wave have dropped to ∼10% of the initial intensity, *i.e.* for a linear distance from the emitter atom up to 2.3λ_IMFP_ (the information depth is usually assumed to be ∼3λ_IMFP_). Given the nearest-neighbor distance of the Re atoms of 0.276 nm, we estimate that in a cluster-type real-space model about 100000 to 400000 atoms [in the sequence (*a*) to (*f*)] contribute significantly to the diffraction pattern. We have found that the XPD results for graphite [an example is shown in Fig. 2(*b*) ▸] can be perfectly described using the Bloch-wave approach to XPD (Fedchenko *et al.*, 2019 ▸). Since the photoelectron wavelength is more than an order of magnitude smaller than the nearest-neighbor distance, phase differences caused by small changes in the lattice structure are ‘amplified’ by this factor. The rich structure in Figs. 2(*b*), 2(*c*), 7 and 8 ▸ indicates that high-resolution small-angle XPD has the potential of being a very sensitive structural tool, in particular due to the inherent element specificity of XPD.

The *valence-band *k*-patterns* in Fig. 5 ▸ have been corrected for an underlying background originating from quasi-elastic phonon scattering. The ratio of non-scattered photoelectrons from the direct transition [as sketched in Fig. 6(*a*) ▸] to all emitted electrons is governed by the Debye–Waller factor. The case of rhenium is favorable because it is heavy (*Z* = 75) and has a high Debye temperature (416 K). Due to its high Debye–Waller factor at 30 K, one expects a fraction of 25% non-scattered photoelectrons for a kinetic energy of 3.5 keV. This background has a spectral distribution given by the matrix-element-weighted density of states (Osterwalder *et al.*, 1990 ▸, 1996 ▸; Herman *et al.*, 1992 ▸; Trehan *et al.*, 1987 ▸; Krüger *et al.*, 2011 ▸), and can be corrected as described by Gray *et al.* (2011 ▸). In addition, this background carries a Kikuchi-type diffraction signature because after phonon scattering the electrons are diffracted at the lattice. In fact, the diffraction pattern of the background is identical to a core-level diffraction pattern taken at the same final-state energy and identical settings of the microscope (*k*-field-of-view, energy). Moreover, also the valence-band patterns themselves show diffraction effects. We found that an efficient way to remove the diffraction modulation of background and true valence-electron signal is pixel-by-pixel division of the as-measured valence-band map by a core-level XPD pattern at the same final-state energy. This procedure was applied to the rhenium valence-band maps shown in Fig. 5 ▸. For more details on the correction procedure and its relation to the Debye–Waller factor, see Babenkov *et al.* (2019 ▸). So far, we have not studied the recoil effect of the photoelectrons as discussed for example by Takata *et al.* (2008 ▸) and Suga & Sekiyama (2014 ▸). Given our energy resolution of 60 meV [with the Si(333) monochromator crystal], it should be easily possible to observe this effect, especially in compound materials or heterogeneous systems containing heavy and light elements.

## 4. Summary and conclusions   

This paper introduces an effective way to cope with the weak signals in hard X-ray angular-resolved photoelectron spectroscopy (HARPES). Full-field momentum imaging (*k*_*x*_,*k*_y_) combined with time-of-flight (τ) parallel energy recording constitute a 3D recording scheme. Parallel acquisition of *I*(*k*_*x*_,*k*_*y*_,τ)-arrays in a *k*-space range with diameters up to 25 Å^−1^ and a useful energy range of ∼8 eV width substantially enhance the phase-space volume recorded simultaneously, in comparison with conventional 2D recording. A special objective lens and retarding zoom optics have been developed, optimized for low aberrations at high energies and *k*-fields of view an order of magnitude larger than recorded by the existing low-energy momentum microscopes. As a special feature this objective lens can be operated with zero extractor field, thus opening the path to samples with corrugated surfaces, small crystallites and 3D micro-/nano-structures and generally to free sample rotation in front of the lens. In conventional momentum microscopes with high extractor field the sample must be essentially planar. The microscope can be operated in real-space imaging mode (PEEM) for checking the surface quality, finding suitable regions of interest on inhomogeneous samples or for observing and positioning of the photon footprint on the surface.

The increase of dimensionality of the recording scheme effectively counteracts the dramatic drop in photoemission cross sections and the increase of electron–phonon scattering with increasing photon energy in the several-keV range. The bottleneck for the performance of a HARPES experiment is the parallel accessible *E*–***k*** phase-space volume. This is different from low-energy ARPES, where intensities are much higher so that the electron detector is the bottleneck.

The performance of instrument and method was elucidated in first experiments at the new dedicated hard X-ray beamline P22 at PETRA III (DESY, Hamburg). *I*(*E*_B_,***k***) data arrays have been recorded at many photon energies in the range 2.6–6 keV. Low sample temperature (∼30 K) ensured a sufficiently high Debye–Waller factor. The large *k*-field-of-view allows recording valence-band *k*-maps that comprise many BZs (19 BZs in the case of Re). Thanks to the high brilliance (10^13^ *h*ν s^−1^ in a spot of ∼20 µm), valence-band signals reached up to several million counts per second in the *d*-band complex of transition metals (Re, Mo). Under such conditions, 3D bulk BZs can be mapped in a tomography-like manner in a few hours by varying the photon energy and exploiting direct transitions into free-electron-like final states. Recording multiple BZs can be utilized for a multiplexing of the intensity. Adding equivalent repeated patterns (exploiting the *k*-space periodicity) is an effective way of intensity enhancement. Moreover, this procedure largely eliminates the matrix-element effect and partly also photoelectron diffraction contributions. Such an enhancement of intensity via ‘large-area recording’ and adding up equivalent band features of several BZs is not only interesting for momentum-microscopy but also for experiments using hemispherical analyzers.

Photoelectrons from the valence band are the fastest and thus easily accessible by a ToF spectrometer, which is inherently a high-pass filter. A crucial question prior to the experiments was whether core-levels are accessible as well. Indeed, XPS of core-levels was possible, as well as fast recording of XPD patterns. The information content of XPS as a sensitive *chemical* probe and XPD as a probe of the *geometric structure* ideally complement the *electronic structure* information of valence-band momentum mapping. XPS survey spectra in terms of wide-range ToF spectra (several hundred eV wide) give information on the chemical state of sample and surface, with a sensitivity in the few-% range. The high-pass characteristics of ToF instruments in combination with the excitation at the photon-pulse period *T* can lead to *temporal aliasing*: electrons with flight times longer or shorter by a multiple of *T* (condition Δτ = ±*nT*) fall into the same time interval causing undesired overlapping signals. Systematic simulations uncovered the conditions under which temporal coincidence of unwanted signals from other core-levels, Auger electrons or signals from higher orders of the monochromator/undulator can occur. We show examples of this effect, *e.g.* a *L*_3_ *MM* Auger signal released by third-order photons with energy as high as *h*ν = 11.25 keV, and discuss various strategies to avoid or suppress such temporal coincidences. The signals from faster electrons can be easily identified by their different focusing properties, suggesting geometric beam blanking as a suppression method.

Full-field core-level XPD patterns at very high angular resolution of ∼0.035° are recorded within minutes. These small-angle diffractograms (polar angular range 0–11° at 6 keV) uncover a new appearance of XPD. The short photoelectron wavelength (more than an order of magnitude smaller than the nearest-neighbor distance) leads to small Bragg angles of few degrees for reflection at the lattice planes perpendicular to the surface. The small wavelength ‘amplifies’ the sensitivity of the interference pattern and is the key factor for the richness in details. Kikuchi bands can be identified in the XPD patterns and provide a *k*-space ruler for analysis of the momentum patterns. The patterns react strongly on changes of photon energy [Fig. 8 ▸, see also Fedchenko *et al.* (2019 ▸)].

In conclusion, the present development towards large-area *k*-imaging at hard X-ray energies facilitates two key applications: mapping of bulk band structures, parallel in several BZs (Figs. 3 ▸ and 5 ▸) enhancing recording efficiency by summing up several equivalent BZs; and fast recording of full-field core-level XPD patterns with Kikuchi-type diffraction signature and many filigrane details (Figs. 7 ▸ and 8 ▸), which provide a sensitive tool probing geometric structure. Structural and electronic information are obtained quasi-simultaneously in an identical setup. This property makes high-resolution hard X-ray *k*-microscopy a powerful tool for real-time observation of structural changes in the lattice, and the correlation with changes of the electronic structure in core-level spectra (*e.g.* changes of local electron density) and the valence-band region. Combined HARPES and hard-XPD recording will be of particular advantage when studying phase transitions or ultrafast dynamics (see Kutnyakhov *et al.*, 2019 ▸).

Further possibilities for future experiments are opened by the field-free imaging mode, being for the first time explored in this paper. This operation [Fig. 2(*c*) ▸] allows free sample rotation in front of the entrance lens. The PEEM mode is helpful in order to keep the photon footprint in the same position during rotation. A further exciting (though not yet proven) future aspect is the possibility to reconstruct the full 4D spectral function from a measurement at a few photon energies or even just a single one, exploiting the curvature of the final-state sphere. Previously, this was done via tomography-like measurements at many photon energies (Medjanik *et al.*, 2017 ▸). The *k*-field curvature along with the large field of view with many BZs leads to the fact that the contour crosses the BZs at different *k*_*z*_ values.

## Supplementary Material

Supplementary movies - see Fig. 6. DOI: 10.1107/S1600577519012773/il5041sup1.avi

Supplementary movies - see Fig. 6. DOI: 10.1107/S1600577519012773/il5041sup2.gif

Supplementary movies - see Fig. 6. DOI: 10.1107/S1600577519012773/il5041sup3.tif
