# Supplementary figures and images for: Progress in HAXPES performance combining full-field k-imaging with time-of-flight recording
Source: J Synchrotron Radiat. 2019 Nov 1;26(Pt 6):1996–2012. doi: 10.1107/S1600577519012773 (PMC6853377; doi:10.1107/S1600577519012773)

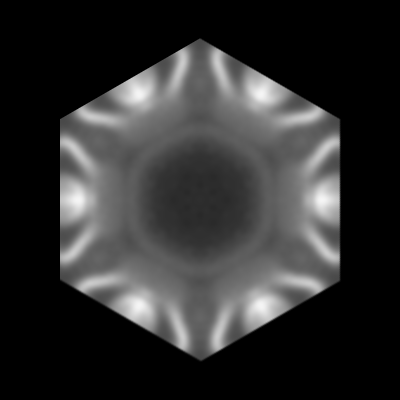

Supplement: Supplementary file 2 [file s-26-01996-sup2.gif]

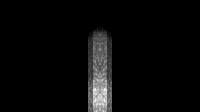

Supplement: Supplementary file 3 [file s-26-01996-sup3.tif]
